# Utilization of rehabilitation services for inpatient with cancer in Taiwan: a descriptive analysis from national health insurance database

**DOI:** 10.1186/1472-6963-12-255

**Published:** 2012-08-16

**Authors:** Heui-Fen Lin, Ying-Tai Wu, Jau-Yih Tsauo

**Affiliations:** 1 School and Graduate Institute of Physical Therapy, College of Medicine, National Taiwan University, Floor 3, No 17, Xuzhou Rd, Taipei, Taiwan; 2 Division of Physical Therapy, Department of Physical Medicine and Rehabilitation, National Taiwan University Hospital, No.7, Chung Shan S. Rd, Taipei, Taiwan; 3 Physical Therapy Center, National Taiwan University Hospital, No.7, Chung Shan S. Rd, Taipei, Taiwan

**Keywords:** Cancer, Rehabilitation, Utilization, Taiwan

## Abstract

**Background:**

Cancer is a major cause of global morbidity and mortality. Since a high prevalence of functional impairments has been observed among cancer patients, rehabilitation has been proposed as a strategy to restore patients’ functional independence. The increasing number of cancer patients combined with a growing need for rehabilitation may result in increased utilization of rehabilitation services. This study aimed to investigate the utilization of rehabilitation services among hospitalized cancer patients in Taiwan between 2004 and 2008.

**Methods:**

Annual admissions and total inpatient expenditures for admissions with a cancer diagnosis were calculated from the National Health Insurance Research Database (NHIRD). Rehabilitation services used by cancer and non-cancer patients, as well as the distributions of rehabilitation service type among the different hospital departments were also analyzed.

**Results:**

The percentages of inpatient admissions with a cancer diagnosis increased from 14.01% to 17.1% between 2004 and 2008. During 2004, 5.25% of all inpatient admissions received rehabilitation services; this percentage increased to 5.62% by 2008. Among cancer admissions, 2.26% to 2.62% received rehabilitation services from 2004 to 2008. By comparison, 5.68% to 6.24% of non-cancer admissions received rehabilitation services during this period. Of the admissions who received rehabilitation services, only 6.44% and 7.96% had a cancer diagnosis in 2004 and 2008, respectively. Sixty-one percent of rehabilitation services were delivered in the departments of orthopedics (25.6%), neurology (14.4%), rehabilitation (11.9%), and neurosurgery (9.2%).

**Conclusions:**

In Taiwan, the utilization of rehabilitation services during hospitalization increased from 2004 to 2008. Although this trend was noted for cancer and non-cancer admissions, the utilization of rehabilitation services was generally greater by non-cancer admissions. Despite the benefits of rehabilitation, the actual rehabilitation needs of cancer patients remain unmet.

## Background

Cancer is a major cause of global morbidity and mortality. Although the incidence of cancer is increasing, improvements in early diagnosis and treatment have led to significantly increased survival rates in recent years. Unfortunately, cancer treatments may result in physical and mental impairment, including dysfunction of the nervous, musculoskeletal, and internal organ systems. Cancer-related fatigue and deconditioning have also been frequently reported as side effects of cancer and cancer treatments. The impairments and symptoms experienced by cancer patients typically contribute to disability and loss of function [1]. Indeed, studies have demonstrated a high prevalence of functional impairments among cancer patients with a wide range of diagnoses and treatment situations [1-8]. In an effort to improve the quality of life of cancer survivors, increasing attention has been given to improving functional recovery following treatment. Rehabilitation has been proposed as a strategy for restoring patients’ functional independence and improving their psychological function [1,8-10]. Moreover, it has been recommended that oncology inpatients receive physical therapy services during their hospital stays to prevent deconditioning [11].

Inpatient rehabilitation has been shown to improve functional status in individuals with disability caused by cancer or its treatment [11-18]. Rehabilitation or exercise intervention programs at acute medical, surgical oncology, or hospice units have been found to be safe and to enhance physical and psychological functioning among hospitalized cancer patients [11,15-17,19-22]. Cancer patients have been found to benefit as much as non-cancer patients from participating in comprehensive rehabilitation programs [23,24]. In response to these programs, patients, families, and even hospital staff reported improved satisfaction with rehabilitation. [11,15,16]. Thus, it is likely that the coming years will see an increased need for the rehabilitation of cancer patients.

The increasing number of cancer patients combined with the growing need for rehabilitation may result in increased utilization of rehabilitation services. Previous studies on rehabilitation needs or utilization among cancer patients, in the form of retrospective chart reviews, questionnaire surveys, or clinician-administered testing, were based on small sample sizes [2-4,6,12-16]. Moreover, information regarding utilization of cancer rehabilitation services in Taiwan is lacking. The differences in utilization patterns of rehabilitation services between cancer and non-cancer patients are also unclear.

Since March 1, 1995, Taiwan has offered a single-payer National Health Insurance (NHI) program that provides universal coverage and equal access to health-care services. By the end of 2008, 99% of the population was enrolled in the program, while 92% of all health-care facilities in Taiwan contracted with the NHI system [25]. The NHI Research Database (NHIRD), a large computerized database provided by the Bureau of National Health Insurance (BNHI) and maintained by the National Health Research Institutes (NHRI, Taiwan), is available to researchers in Taiwan for research purposes. The NHIRD contains registration files and original claim data for reimbursement of NHI programs [26]. As such, it offers researchers a nationwide representative database of the health-care system in Taiwan.

By extracting and analyzing data from the NHIRD, the present study aimed to investigate utilization of rehabilitation services among hospitalized cancer patients in Taiwan between 2004 and 2008.

## Methods

### Data sources and data collection

Data collected during 2004 and 2008 and made available by Taiwan’s NHIRD were used for analysis. Specifically, data on inpatient expenditures by admissions (retrieved from the DD file) and related records of detailed inpatient orders (retrieved from the DO file), were accessed. The DD file was extracted from the original NHI claim data using a sampling rate of 5%.

Admissions with a cancer diagnosis were identified using the following: an International Classification of Diseases, Ninth Revision, Clinical Modification (ICD-9-CM) diagnostic code consisting of 140 to 239 as the first three digits, other cancer-related codes consisting of A08 to A17 as the first three digits, and cancer surgery-related codes from V57 to V58 as the first three digits. If the case did not meet any of the above criteria, the admission was categorized as “non-cancer.”

When the rehabilitation fee within the claim data was not equal to zero, the case was identified as “using rehabilitation services.” Otherwise, the case was classified as requiring “no rehabilitation.” According to the scope of NHI benefits, the rehabilitation therapy fee included services related to rehabilitative examination and treatment, including physical, occupational, communication, and psychosocial therapy. Details of rehabilitation services were obtained from the linked DO dataset.

Because the data for identifying the patient or institution had been scrambled cryptographically by the NHIRD, this study was exempt from the requirement of institutional review board approval.

### Statistical analysis

The annual number of admissions with a cancer diagnosis and the percentages of total admissions that were due to cancer cases were calculated. Rehabilitation service use among admissions with and without a cancer diagnosis and the distributions of rehabilitation service delivery among hospital departments were analysed. The annual admission numbers and medical expenditures were multiplied by a factor of 20 to account for the 5% systematic sampling data sets. Data reduction and descriptive statistics were performed using SAS software, version 9.1 (SAS Institute, Inc, Cary, NC, USA).

## Results

### Annual admissions with cancer diagnosis and utilization of rehabilitation services

Approximately 3 million hospital admissions occurred per annum between 2004 and 2008. As indicated in Table 1, the percentages of admissions that claimed rehabilitation services in 2004, 2005, 2006, 2007, and 2008 were 5.25%, 5.27%, 5.37%, 5.57%, and 5.62%, respectively. The percentage of admissions with a cancer diagnosis was 14.01% in 2004, 14.94% in 2005, 15.61% in 2006, 16.50% in 2007, and 17.10% in 2008. In 2004, 6.44% of the admitted patients that utilized rehabilitation services had a cancer diagnosis; by 2008 this percentage increased to 7.96%. The utilization rates of rehabilitation services were much lower for cancer versus non-cancer admissions (Table 1).

**Table 1:** Annual numbers of admissions from 2004 to 2008

	**2004**	**2005**	**2006**	**2007**	**2008**
Total admissions*	3020.2	2975.9	2921.1	2968.8	3046.6
Cancer admissions *	423.0	444.6	454.6	489.8	520.8
(% of total admissions)	(14.01%)	(14.94%)	(15.61%)	(16.50%)	(17.10%)
Admissions received rehabilitation services*	158.4	156.8	156.5	165.4	171.3
(% of total admissions)	(5.25%)	(5.27%)	(5.37%)	(5.47%)	(5.62%)
Non-cancer admissions received rehabilitation services*	148.2	146.7	156.5	152.9	157.7
(% of non-cancer admissions)	(5.71%)	(5.80%)	(5.68%)	(6.17%)	(6.24%)
Cancer admissions received rehabilitation services*	10.2	10.1	11.3	12.5	13.6
(% of cancer admissions)	(2.41%)	(2.26%)	(2.48%)	(2.56%)	(2.62%)
(% of admissions received rehabilitation services)	(6.44%)	(6.42%)	(7.21%)	(7.58%)	(7.96%)

* unit: thousand.

Sixty-one percent of all rehabilitation services were delivered to just four hospital departments: orthopedics (25.6%), neurology (14.4%), rehabilitation (11.9%), and neurosurgery (9.2%). A similar distribution of rehabilitation services according to different departments was observed during the years assessed. Physical therapy (PT) and occupational therapy (OT) accounted for 62% and 32% of all rehabilitative treatments offered in 2008.

### Annual medical expenditures and annual claims for inpatients rehabilitation services

As described in Table 2, annual medical expenditures for inpatient care amounted to 145.9 billion points in 2004 and 159.9 billion points in 2008. According to a medical claims review, the reimbursement from NHI for one point is approximately 0.8 to 1.0 NTD. Accordingly, annual inpatient medical expenditures increased 9.6% from 2004 to 2008. Annual inpatient medical care for cancer admissions accounted for 18.7% of total inpatient medical expenditures in 2004, and increased to 22.2% by 2008. Annual claims for inpatient rehabilitation services ranged from 923 to 994 million points between the years of 2004 to 2008, and accounted for 0.6% of total inpatient medical expenditures. The average inpatient medical expenditure per admission gradually increased from 48,304 points in 2004 to 52,475 points in 2008. The average medical expenditure for admissions with a cancer diagnosis was higher than those with a non-cancer diagnosis. However, the average rehabilitation fee was lower for admissions with a cancer versus a non-cancer diagnosis (Table 3). In fact, the average rehabilitation fee for admissions with a cancer diagnosis was approximately 706 points lower than that for non-cancer admissions. The average number of rehabilitation service units for each admission was between 10.6 and 11.4 for cancer admissions, and between 12.1 and 13.1 units for non-cancer admissions (Table 3).

**Table 2:** Annual medical expenditure of inpatient care from 2004 to 2008

	**2004**	**2005**	**2006**	**2007**	**2008**
Total admissions^*^	145.89	150.24	150.11	152.24	159.87
Cancer admissions^*^	27.28	28.74	30.83	31.97	34.59
(% of total admissions)	(18.70%)	(19.13%)	(20.54%)	(21.00%)	(22.20%)
Non-cancer admissions^*^	118.61	121.50	119.28	120.27	125.28
Rehabilitation services^*^	0.95	0.92	0.94	0.93	0.99
(% of total admissions)	(0.65%)	(0.61%)	(0.63%)	(0.61%)	(0.62%)

*Unit: billion points; 1 point was reimbursed about 0.8-1.0 NTD.

**Table 3:** Average medical expenditure, rehabilitation fee, and rehabilitation service units

	**2004**	**2005**	**2006**	**2007**	**2008**
Average medical expenditure*					
Cancer admissions	64,492	64,653	67,806	65,269	66,419
Non-cancer admissions	45,668	47,999	48,538	48,514	49,600
Average rehabilitation fee*					
Cancer admissions	5,418	5,245	5,190	5,088	5,114
Non-cancer admissions	6,021	5,927	6,083	5,696	5,859
Average rehabilitation service units^*^					
Cancer admissions	11.4	11.3	11.0	10.6	10.8
Non-cancer admissions	13.1	12.7	12.8	12.1	12.3

*Unit: point.

### Rehabilitation utilization among cancer and non-cancer admissions, and the proportion of admissions with a cancer diagnosis by department in 2008

**Figure 1:**
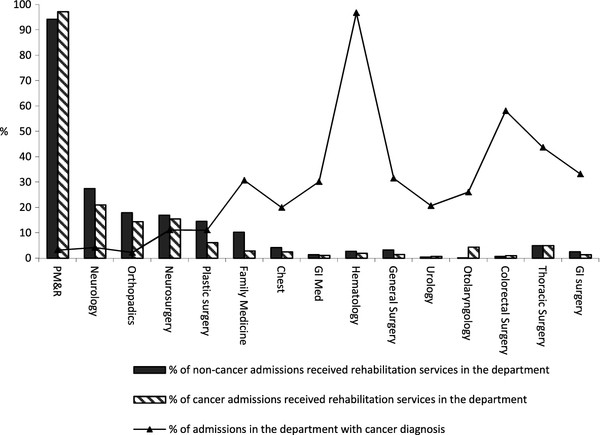
Rehabilitation utilization rates by departments among cancer and non-cancer admissions by year 2008.

Figure 1 shows the proportion of admissions with and without a cancer diagnosis that received rehabilitation services in 2008 according to the hospital department. Five departments with relatively higher rehabilitation utilization and 10 departments with relatively higher percentage of admissions with a cancer diagnosis were chosen for illustration. Relatively higher rehabilitation utilization rates than other departments were noted in rehabilitation, orthopedics, neurology and neurosurgery departments in spite of the cancer diagnosis. Cancer and non-cancer admissions in the department of family medicine had a higher rehabilitation utilization rate (2.87% and 10.29%, respectively) than the average for the entire data sample of cancer and non-cancer admissions (2.62% and 6.24%, respectively). As illustrated in Figure 1, utilization of rehabilitation services was relatively low in the other departments. Above-average rehabilitation service utilization rates were found among cancer admissions being seen at the departments of otolaryngology (4.37%) and thoracic surgery (5%).

The department-specific components of rehabilitative services according for cancer patients receiving rehabilitation services in 2008 were also examined (see Additional file 1). PT accounted for more than 70% of the rehabilitation components, while speech therapy (ST) accounted for less than 5% among most departments. However, there were a few department-specific exceptions in the distributions of rehabilitative services. For instance, the proportions of PT, OT, and ST accounted for 49.02%, 40.93%, and 10.05%, respectively, of rehabilitative services offered in the PM&R department. In department of neurosurgery, PT, OT, and ST accounted for 65.35%, 30.60%, and 4.05% of rehabilitative services offered, respectively. As for department of family medicine, PT provided half of the rehabilitation components (53.01%) and another half by OT (46.18%).

## Discussion

This study used data from a nationwide insurance registration to analyse the utilization pattern of inpatient rehabilitation services in Taiwan, with particular focus on patients diagnosed with cancer. We found a trend of increasing admission of patients with a cancer diagnosis from 2004 to 2008, as well as a trend of increasing utilization of rehabilitative services by cancer patients during hospitalization. This latter observation may indicate a growing awareness of the rehabilitative needs of cancer patients. The increase of cancer admissions claiming rehabilitation services was in line with the increase of admissions of cancer patients. However, the utilization of rehabilitation services was much lower among cancer versus non-cancer admissions along with much lower rehabilitation utilization rate and fewer rehabilitative service.

The utilization of rehabilitation services among hospitalized cancer patients in our study was far below that reported in the literature. The rehabilitation needs of 55 patients admitted to a medical oncology unit were examined by Movsas et al. [6], who reported that 48 (87%) of those patients had rehabilitation needs on admission. Similarly, the medical records of 100 patients with newly diagnosed lung cancer were reviewed by Podnos et al. [4]. In this study, a total of 114 referrals involving 64 patients were made for supportive services. Thirty-one percent of the referrals were for pulmonary rehabilitation, while 19% were for PT or OT. According to another study, of the 100 patients discharged from a palliative-care unit, 37 received a formal PT assessment, and 18 underwent PT [18].

The apparent underutilization of rehabilitative services in this study may be explained by a failure of acute-care staff to identify functional impairments, lack of appropriate rehabilitation referral, lack of awareness of rehabilitation services, and lack of knowledge about rehabilitation services among family members [5,6]. Underutilization of rehabilitation services may also result from the medical complexity of cancer cases in acute-care settings. In a nationwide Japanese survey, 50.8% of institutions stated an “absence of prescriptions for rehabilitation by attending physicians” as the reason for the delayed introduction of rehabilitation for cancer patients; other reasons included insufficient staff (30.4%) and lack of preparation of the institution and necessary facilities (27.1%) [27]. These barriers can be overcome by educational efforts and by promoting interdisciplinary cooperation of clinical staff in oncology [6,17].

The present study found that rehabilitation services were delivered predominantly within the orthopedic, neurology, rehabilitation, and neurosurgery departments regardless of a cancer diagnosis. This finding was not surprising given the scope of traditional physical medicine and rehabilitation. Another important reason for this observation may be specific to Taiwan; according to the reimbursement regulation of the NHI program, only physicians from these departments may directly request PT for their patients. Other physicians had to consult a physiatrist to request rehabilitation services. High caseloads, limited availability, and lack of professionals specialized in cancer rehabilitation may also contribute to low utilization of rehabilitative services in other departments with a high prevalence of cancer patients, such as hematology/oncology.

We noted a discrepancy in the rate of rehabilitative service utilization between cancer and non-cancer patients in the department of plastic surgery. Rehabilitation services for patients in the department of plastic surgery predominantly consisted of OT for splints, which accounted for 59% of total rehabilitation service units for the department. Conversely, a very small number of cancer patients admitted to this department received rehabilitation services.

Above-average rehabilitation utilization rates were found among cancer admissions in the departments of family medicine, otolaryngology, and thoracic surgery. Similar rates of rehabilitation services utilization were noted for cancer and non-cancer patients (5%) admitted to the thoracic surgery department. This may indicate a similar use of pulmonary rehabilitation by both cancer and non-cancer patients. Most hospitals place their hospice-care unit in the family medicine department. The percentage of cancer admissions to the family medicine department increased from 25% to 31% between 2004 and 2008, while the rehabilitation services utilization rate of cancer admissions increased from 2.03% to 2.87% between 2004 and 2008. The growth rate of cancer patients receiving rehabilitation (41%) was much greater than that of cancer patients in the department (24%). This finding may suggest increasing per-patient utilization of rehabilitation services in the hospice-care unit. In the otolaryngology department, 91% of the rehabilitation services were used by cancer patients. The utilization rate of rehabilitation services by cancer patients in the otolaryngology department increased from 3.28% in 2004 to 4.37% in 2008. The increased utilization of rehabilitation services in this group may be explained by the fact that head and neck cancer are relatively common in Taiwan.

The NHIRD has a number of inherent limitations, including a lack of clinical, demographic, and disease severity data (e.g. cancer stages), and other important outcome variables. Thus, we cannot assess the outcomes or effects of rehabilitation services, or the correlation of disease severity and rehabilitation service use. Cancer admissions were identified from admission diagnoses in our study. We could not determine whether or not the cancer patients were newly diagnosed, or whether or not they were admitted for cancer-related problems. According to the Computer-Processed Personal Data Protection Law and related regulations of BNHI and NHRI, data in NHIRD that could be used to identify patients or care providers is scrambled before being released to each researcher. Therefore, we could not compare the rate of rehabilitation service utilization across different hospital settings.

The data used in our study were cross-sectional in nature. Thus, longitudinal study of rehabilitation service utilization following cancer diagnosis is warranted. A comparison of rehabilitation utilization patterns between inpatient and ambulatory care is suggested for further analysis.

## Conclusions

This study documented a rise in the utilization of rehabilitation services during hospitalization by cancer patients in Taiwan. However, the utilization of inpatient rehabilitation services was relatively low among cancer as compared with non-cancer admissions. The distributions of rehabilitation service types among different departments vary between cancer and non-cancer admissions. Further research regarding the delivery of services to meet cancer-specific rehabilitation needs is warranted.

## Abbreviations

NHIRD: National Health Insurance Research Database; NHI: National Health Insurance; BNHI: Bureau of National Health Insurance; NHRI: National Health Research Institutes; OT: Occupational therapy; PT: Physical therapy; ST: Speech therapy; PM&R: Physical medicine and rehabilitation; GI Med: Gastrointestinal medicine; GI surgery: Gastrointestinal surgery.

## Competing interests

All authors declare no competing interests.

## Authors’ contributions

HFL designed and conducted the study, obtained, analysed and interpreted data, and drafted the manuscript. JYT assisted in study design, data analysis, and data interpretation, and provided critical revision of the manuscript for important intellectual concepts. YTW assisted in study design, data interpretation, and manuscript writing. All authors have approved the final submitted manuscript.

## Pre-publication history

The pre-publication history for this paper can be accessed here:

http://www.biomedcentral.com/1472-6963/12/255/prepub

## Supplementary Material

Additional file 1 The department-specific components of rehabilitative services in 2008. Click here for file
